# Babesiosis concurrent with multiple abscesses from *Staphylococcus aureus* infection: A case report

**DOI:** 10.1016/j.heliyon.2023.e18563

**Published:** 2023-07-21

**Authors:** Dongming Xu

**Affiliations:** Department of Emergency Medicine, Beijing Friendship Hospital, Capital Medical University, Beijing 100050, China

**Keywords:** Babesiosis, *Staphylococcus aureus*, Abscess, Case report

## Abstract

**Background:**

Babesiosis is a tick-borne illness. These patients may have signs of a systemic inflammatory response, but abscess formation is unusual. Multiple abscesses in a patient with confirmed babesiosis is very rare, so concurrent infection by another pathogen should be considered.

**Case presentation:**

We report a 42-year-old male patient who had fever, chills, joint pain, abdominal pain, and altered mental status after a possible tick bite on his right foot while fishing in a river. The laboratory tests, including a blood smear, suggested babesiosis. Imaging studies showed multiple brain and spleen abscesses due to *Staphylococcus aureus* based on the results of a blood culture and next-generation sequencing. The patient eventually recovered after treatment with azithromycin, fosfomycin, and vancomycin.

**Conclusion:**

Concurrent bacterial infection can occur in a patient with babesiosis. Additional tests should be performed when a babesiosis patient presents with signs inconsistent with *Babesia* infection. Prompt and appropriate treatment is necessary and may be life-saving for these patients.

## 1 Introduction

Babesiosis is zoonotic infection caused by protozoan of the genus *Babesia* and has an estimated global prevalence of 2.2% [1,2]. Ticks are mostly responsible for *Babesia* transmission. *Babesia microti* is the most common *Babesia* species and *Ixodes ricinus* is the most common tick species [3,4]. Except for occasional reports of human-to-human transmission by contaminated blood, organ transplantation, or maternal-fetal transmission [5], babesiosis is mostly due to transmission by ticks that are obligate surface parasites [6]. During a tick bite, the tick stabs its stinging limbs and infraoral plate into the host skin while sucking blood, with its mouthparts typically firmly fixed onto the skin. Inappropriate removal of the tick is likely to leave mouthparts in the skin and lead to a possible infection [7]. Infected patients can be asymptomatic, can experience a systemic inflammatory reaction, and can sometimes even develop multiple organ failure [8]. Infection by *Babesia* is confirmed using a peripheral blood smear examination or the polymerase chain reaction [9]. *Babesia* infection can occur concurrently with other tick-borne illnesses, such as Lyme disease and anaplasmosis [[10], [11], [12]].

*Staphylococcus aureus* is a Gram-positive bacterium that is a human commensal organism. *S. aureus* usually causes skin and soft tissue infections, but can also enter the bloodstream from broken skin and cause systemic infection and formation of abscesses [13]. Inappropriate or inadequate treatment of an *S*. *aureus* infection can lead to sepsis and multiple organ failure.

Most previous research on coinfections in patients with tick-borne illnesses described infections by two or more different tick-borne pathogens. These coinfections could present a significant diagnostic and treatment challenge [14]. Here, we report a patient with coinfection by *Babesia* and *S. aureus*, an opportunistic pathogen. We show that a patient who presents with tick-borne illness could also have coinfection by a common opportunistic bacterial pathogen. Clinicians should therefore be alert to the possibility of coinfection by a common opportunistic pathogen in a patient who presents with a tick-borne illness.

### 1.1 Case presentation

A 42-year-old local male resident presented to Beijing Friendship Hospital on July 27, 2022 and reported fever, chills, and joint pain. He denied any history of medical or psychiatric problems, as well as any significant family history. Seven days before presentation, the patient went fishing at a nearby river. At that time, he noticed an unknown small insect attached to his right foot; he used his fingers to remove the insect and then threw it away. Six days before presentation, he began to experience an intermittent fever (highest body temperature, 39 °C) with occasional chills, and had pain in the bilateral wrists, right hip, left knee, and abdomen. Two before presentation, he started to develop slurred speech. One day before presentation he complained of headache and blurred vision, appeared restless, irritable, and confused, and experienced loss of consciousness.

**Fig. 1:**
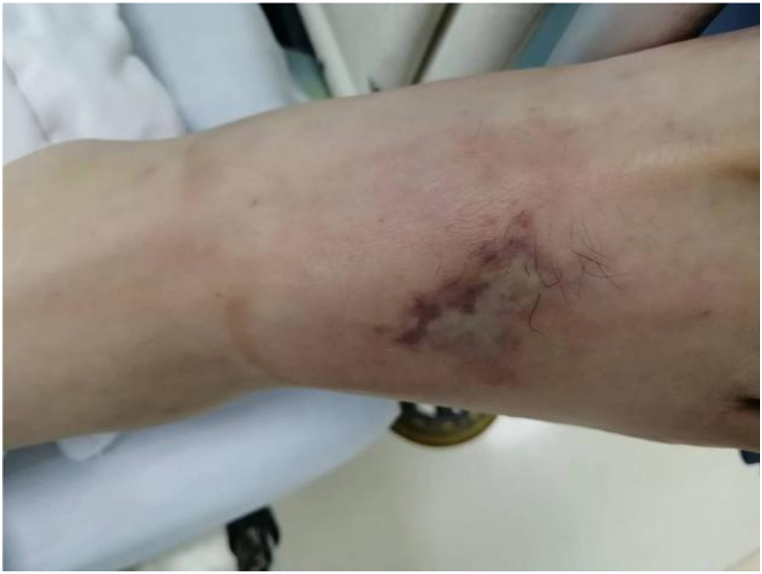
Ecchymosis of the right foot, showing the location of the insect bite.

At admission, the patient had a temperature of 39 °C, blood pressure of 126/84 mmHg, heart rate of 104 beats/min, and respiratory rate of 20 times/min. He appeared confused and disoriented, and had an ecchymosis on the dorsal side of his right foot (Fig. 1). His lung sounds were clear, but heart auscultations indicated systolic murmurs at the apex of the heart. Abdominal palpation indicated slight tenderness in the left upper quadrant.

**Fig. 2:**
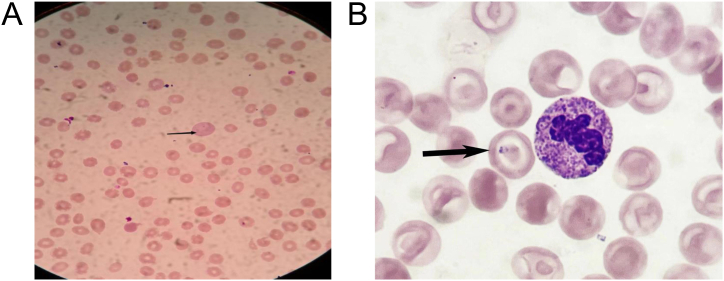
Presence of *Babesia* in a blood smear (Giemsa staining), in which a pale blue cytoplasm surrounds a vacuole with one or two clumps of chromatin, mainly at one end of the cytoplasm. (A, light microscopy at 1000 × . B, light microscopy with immersion oil at 1000 × . Zeiss, Germany).

The laboratory blood test results are shown in Table 1. A blood smear test was performed by spreading a thin layer of a peripheral blood sample on a slide and staining with the Giemsa solution (Solaribio Science & Technology Co., Ltd., Beijing, China). The results showed the characteristic *Babesia* rings, in which pale blue cytoplasm surrounded a vacuole with clumps of chromatin, mainly at one end of the cytoplasm (Fig. 2). These results support a diagnosis of babesiosis.

**Table 1:** Blood laboratory test results.

Laboratory test	Result	Normal range
White blood cell count	12.6 × 10^9^/L	3.5–9.5 × 10^9^/L
Hemoglobin	16.4 g/L	130–175 g/L
Platelet count	49 × 10^9^/L	125–350 × 10^9^/L
Urea nitrogen	11.6 mmol/L	3.1–8.0 mmol/L
Creatinine	97.0 mmol/L	41–111 mmol/L
Total bilirubin	40.1 μmol/L	3.4–21.0 μmol/L
Direct bilirubin	24.9 μmol/L	0–6.8 μmol/L
Alanine transaminase	50 U/L	9–50 U/L
Aspartate aminotransferase	102.5 U/L	15–40 U/L
Procalcitonin	32.9 ng/mL	<0.1 ng/mL
Prothrombin time	12.4 s	9.6–13.5 s
Activated partial thromboplastin clotting time	26.2 s	21–34 s
International normalized ratio	1.45	0.8–1.2

**Fig. 3:**
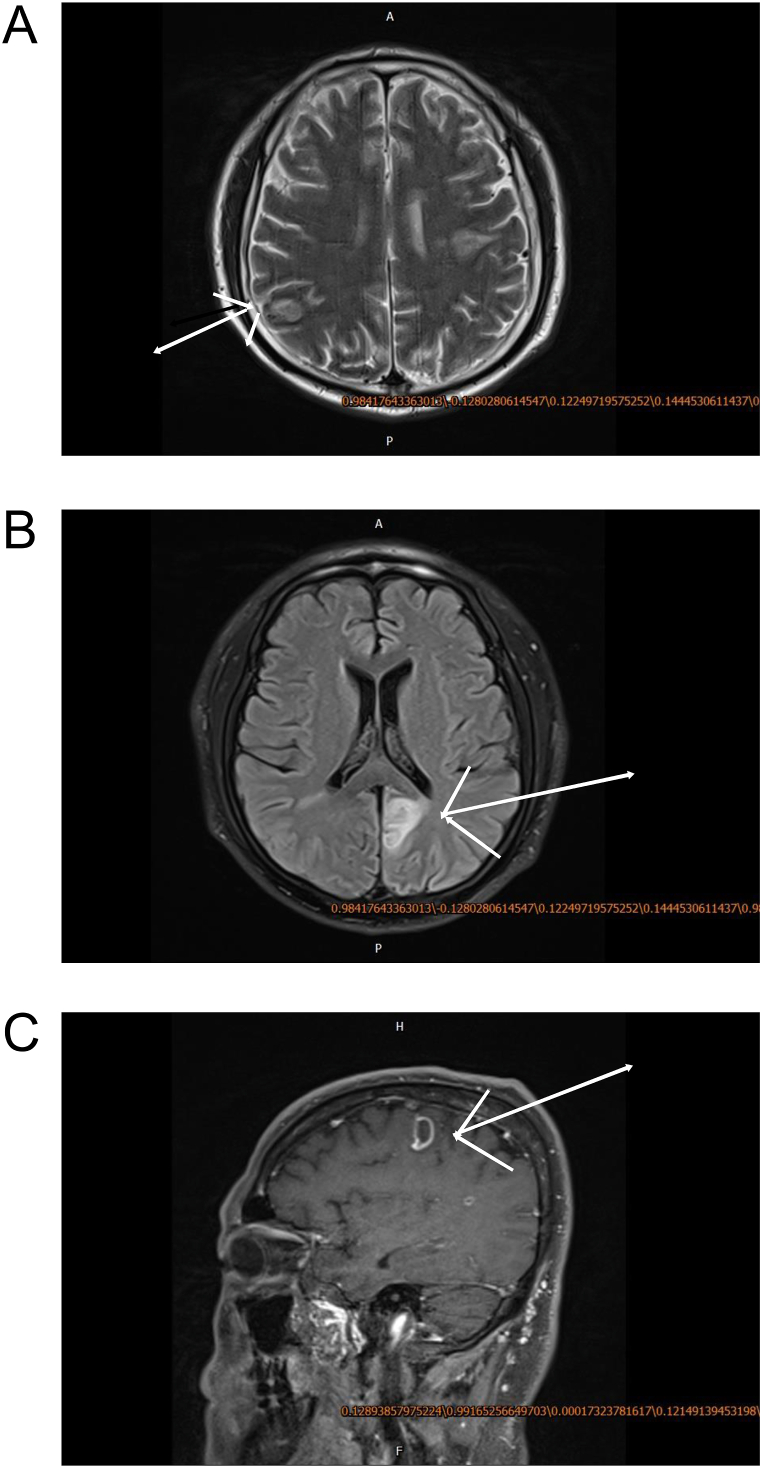
Magnetic resonance imaging of the head with multiple ring-enhanced abscesses. A. Axial position. B. Coronal position. C. Sagittal position.

However, an abdominal ultrasound examination (Mindray, BC-8, Shenzhen, China) showed a 5.8 × 2.8 cm splenic abscess, and magnetic resonance imaging (MRI, Siemens 3.0 T, Germany) indicated brain abscesses (Fig. 3), although an echocardiogram indicated no obvious vegetation. A blood culture led to the identification of *S. aureus* (antibiotic sensitivity data in Table 2), and next-generation sequencing of a blood sample indicated *S. aureus* sequence 1 (with a coverage of 50%, average of 2079 reads, and positive reference coverage range ≥20% (Seq&Treat Medical Technology Co., China) [15]. Finally, we modified the diagnosis as babesiosis with *S. aureus* splenic and brain abscesses. Considering the presence of heart murmurs, we also suspected infective endocarditis.

**Table 2:** Antibiotic sensitivity analysis for the *S. aureus* isolate.

Antibiotic	Minimum inhibitory concentration (μg/mL)	Sensitive or Resistant
Vancomycin	≤0.5	Sensitive
Gentamicin	≤0.5	Sensitive
Levofloxacin	0.25	Sensitive
Clindamycin	0.25	Sensitive
Linezolid	2.0	Sensitive
Ceftaroline	0.25	Sensitive
Oxacillin	0.5	Sensitive
Sulfamethoxazole and trimethoprim	≤10	Sensitive
Daptomycin	0.5	Sensitive
Erythromycin	≤0.25	Sensitive
Moxifloxacin	≤0.25	Sensitive
Rifampicin	≤0.5	Sensitive
Tigecycline	≤0.12	Sensitive
Penicillin	≥0.5	Resistant
Teicoplanin	≤0.5	Sensitive

We administered azithromycin (0.5 g daily), fosfomycin (8 g every 12 h), and vancomycin (0.4 g every 12 h) after admission, and his body temperature gradually decreased. As these treatments continued, a second peripheral blood smear at day 10 after admission showed no *Babesia* rings. Subsequent complete blood cell counts showed that the white blood cell count gradually returned to the normal range; the hemoglobin level decreased, reached a nadir (7.3 g/dL) at 3 weeks after hospital admission, and then gradually increased. A second cranial MRI showed decreased sizes of the intracranial lesions and a second abdominal ultrasound showed that the splenic abscess decreased in size to 4.3 × 3.4 cm. On day 30 after admission, the patient had no clinical symptoms. Revaluation of his clinical state and laboratory tests at that time indicated a return to normal. Antibiotics were stopped and the patient was discharged and followed up in the clinic.

## 2 Discussion and conclusions

When a tick bites the human body, its forelimbs and mouthparts tightly attach to the skin. Most ticks carry a variety of bacteria and viruses, and many of them can cause infections at the site of skin entry [16]. The estimated global prevalence of *Babesia* is 2.2%, and the highest infection rate is in North America.

*Babesia* is commonly transmitted by ticks, but most people with tick bites do not develop babesiosis. There are hundreds of *Babesia* species worldwide, and most of them only infect domesticated and wild animals. However, *B. microti*, *B. divergens*, *B. venatorum*, *B. duncani*, *Babesia* sp. *MO1*, and *Babesia* sp. *EU1* can infect humans [8]. *Babesia* infects red blood cells, and the most direct evidence for *Babesia* infection is from a microscopic examination of a blood smear, which typically shows multiple circular bodies within the red blood cells, without pigment particles. In addition, a peripheral blood smear of patients with babesiosis may show a few red blood cells containing protozoa. The clinical symptoms of *Babesia* infection include high fever, chills, sweating, headache, fatigue, muscle ache, joint pain, and enlargement of the liver, spleen, and lymph nodes. Neurological symptoms, such as depression, anxiety and panic, memory change, and lethargy, may also occur. A previous report found that *Babesia* infection could cause two types of anemia: non-autoimmune hemolytic anemia and autoimmune hemolytic anemia [17]. The former is caused by direct *Babesia* invasion and destruction of red blood cells; the latter is due to the production of autoantibodies against red blood cells, usually occurs after resolution of the infection, and is associated with certain comorbidities, such as asplenia, sickle cell disease, and malignancy. Both types of anemia can lead to increased levels of direct bilirubin, alkaline phosphatase, aspartate aminotransferase, alanine aminotransferase, and lactate dehydrogenase. In addition, thrombocytopenia and acute renal failure may occur. If a patient does not receive active and prompt treatment, organ failure and death are possible due to the accumulation of toxins and hypoxia. Patients with diminished immune function can rapidly progress to multiple organ failure or even death following *Babesia* infection [18,19].

Our patient had evidence of concurrent infection by *Babesia* and *S. aureus*. The *S. aureus* infection was responsible for the brain abscess, spleen abscess, and infective endocarditis. Our MRI examinations provided direct evidence of the brain abscesses, with multiple infectious foci. The abdominal ultrasound results showed a splenic abscess, and the heart murmur in the background of systemic infection suggested infective endocarditis. Because the blood culture was positive for *S. aureus* and *Babesia* infection does not cause abscess formation, we believe that all the abscesses were due to the *S. aureus* infection. Patients with *S. aureus* brain abscesses typically have significantly increased counts of white blood cells and neutrophils, and increased intracranial pressure. In particular, the white blood cell count is often in the range of 1000 to 10,000 × 10^6^/L, with a predominance of polymorphonuclear granulocytes, and the cerebrospinal fluid culture may be positive for bacteria [20,21]. T1-weighted MRI results typically show that the subarachnoid space is asymmetric, with slightly higher signal intensity and irregular enhancement. The signal intensity of the meninges and cerebral cortex are increased on T2-weighted MRI. Proton density imaging often shows that the exudate from the basal cistern has a relatively high signal intensity compared with the adjacent brain parenchyma. At the later stage of disease, some CT or MRI images may show ependymitis, subdural effusion, and local brain abscess.

The co-occurrence of a brain abscess from *S. aureus* and a *Babesia* infection is extremely rare. Our patient reported a bite by an unknown insect and presented with signs of systemic infection. The laboratory tests and blood smear results were consistent with *Babesia* infection. We therefore suspected that the unknown insect was a tick, the common vector of *Babesia*. A previous study reported patients with *Babesia* infections in the same local area [22]. In northern China, *Babesia venatorum* is the main species, and the main tick vectors are *Haemaphysalis longicornis*, *Rhipicephalus haemaphysialoids*, and *Ixodes persulcatus* [23]. The imaging studies showed multiple abscesses in the brain and spleen that were likely caused by *S. aureus* infection. This pathogen might have been from an insect bite on the skin that happened 7 days before hospital presentation. The prevention of *Babesia* infection requires minimizing skin exposure. The wearing of shirts with long sleeves and long pants may provide protection during outdoor activities. In addition, appropriate care of skin wounds may decrease the risk of *S. aureus* infection. The presence of concurrent *S. aureus* and a *Babesia* infections require treatment with a combination of antibiotics, and these could potentially lead to adverse or unexpected events. In addition to the gastrointestinal side effects, bone marrow suppression may occur. Our patient developed anemia after the antibiotic treatment, suggesting the importance of monitoring adverse effects from multiple antibiotic treatments in these patients. In addition, an abscess could possibly rupture and cause disseminated infection. Rapid changes of clinical symptoms should prompt careful re-evaluations in these patients.

Previous studies also reported coinfections in patients with tick-borne illnesses. However, these studies focused on coinfections by two or more different tick-borne pathogens. These pathogens could have synergistic, indifferent, or antagonistic interactions with each other and the host, and can be a significant challenge for diagnosis and treatment [14]. Here, we report coinfection by tick-borne pathogen and a common opportunistic bacterial pathogen, *Staphylococcus aureus*. These two infections seemed to have no direct connection to each other, although the broken skin caused by the tick bite could certainly increase the risk of infection by bacteria that commonly colonize the skin. If unrecognized, these coinfections could lead to inadequate antibiotic coverage and poor outcome. The key strengths of our study were that we used next-generation sequencing for identification of *S. aureus* and we used abdominal ultrasound for analysis of the splenic abscess. A limitation of our study was that we did not check his immune status or occupational background, which might have predisposed him for serious infectious disease. We will pay attention to these issues in our future clinical practice.

In conclusion, we report a unique case of coinfection by *Babesia* and *S. aureus* to remind clinicians that they should perform additional tests to look for other causes of illness when they encounter a babesiosis patient who presents with additional clinical findings that are inconsistent with infection by *Babesia* alone. Prompt and appropriate treatment is necessary, and may be life-saving.

## Ethics approval and consent to participate

This study was approved by the Ethics Committee of the Beijing Friendship Hospital, Capital Medical University and was conducted in accordance with the principles of the Declaration of Helsinki. Written informed consent was obtained from the individual participant included in the study.

## Consent for publication

Written informed consent was obtained from the patient for publication of this case report and any accompanying images. A copy of the written consent is available for review by the Editor of this journal.

## Author contribution statement

All authors listed have significantly contributed to the investigation, development and writing of this article.

## Data availability statement

Data included in article/supp. Material/referenced in article.

## Declaration of competing interest

The authors declare that they have no known competing financial interests or personal relationships that could have appeared to influence the work reported in this paper.

## List of abbreviations

MRI: magnetic resonance imaging
BIL: direct bilirubin
ALP: alkaline phosphatase
AST: aspartate aminotransferase
ALT: alanine aminotransferase
LDH: lactate dehydrogenase
